# Differential Role of Threonine and Tyrosine Phosphorylation in the Activation and Activity of the Yeast MAPK Slt2

**DOI:** 10.3390/ijms22031110

**Published:** 2021-01-23

**Authors:** Gema González-Rubio, Ángela Sellers-Moya, Humberto Martín, María Molina

**Affiliations:** Departamento de Microbiología y Parasitología, Facultad de Farmacia, Instituto Ramón y Cajal de Investigaciones Sanitarias (IRYCIS), Universidad Complutense de Madrid, Plaza de Ramón y Cajal s/n, 28040 Madrid, Spain; gemagonzalezrubio@ucm.es (G.G.-R.); angselle@ucm.es (Á.S.-M.)

**Keywords:** cell wall integrity, MAPKs, signaling, Slt2, Msg5, phosphorylation, monophosphorylation, Phos-tag

## Abstract

The Mitogen-Activated Protein Kinase (MAPK) Slt2 is central to signaling through the yeast Cell Wall Integrity (CWI) pathway. MAPKs are regulated by phosphorylation at both the threonine and tyrosine of the conserved TXY motif within the activation loop (T190/Y192 in Slt2). Since phosphorylation at both sites results in the full activation of MAPKs, signaling through MAPK pathways is monitored with antibodies that detect dually phosphorylated forms. However, most of these antibodies also recognize monophosphorylated species, whose relative abundance and functionality are diverse. By using different phosphospecific antibodies and phosphate-affinity (Phos-tag) analysis on distinct Slt2 mutants, we determined that Y192- and T190-monophosphorylated species coexist with biphosphorylated Slt2, although most of the Slt2 pool remains unphosphorylated following stress. Among the monophosphorylated forms, only T190 exhibited biological activity. Upon stimulation, Slt2 is first phosphorylated at Y192, mainly by the MAPKK Mkk1, and this phosphorylation is important for the subsequent T190 phosphorylation. Similarly, dephosphorylation of Slt2 by the Dual Specificity Phosphatase (DSP) Msg5 is ordered, with dephosphorylation of T190 depending on previous Y192 dephosphorylation. Whereas Y192 phosphorylation enhances the Slt2 catalytic activity, T190 is essential for this activity. The conserved T195 residue is also critical for Slt2 functionality. Mutations that abolish the activity of Slt2 result in a high increase in inactive Y192-monophosphorylated Slt2. The coexistence of different Slt2 phosphoforms with diverse biological significance highlights the importance of the precise detection of the Slt2 phosphorylation status.

## 1. Introduction

Mitogen-Activated Protein Kinase (MAPK) pathways are evolutionarily conserved transduction modules in eukaryotes that convert a variety of disparate signals into cellular responses, allowing cells to quickly adapt to changing environmental conditions and promoting cell growth and survival. The canonical MAPK cascade consists of a core module of three protein kinases that are sequentially activated by phosphorylation, termed MAPK kinase kinase (MAPKKK, MAP3K, or MEKK), MAPK kinase (MAPKK, MAP2K, or MEK), and MAPK [1]. Four conventional MAPK cascades have been defined in mammalian cells and named according to their corresponding MAPK component. These are extracellular signal-regulated kinases 1 and 2 (ERK 1/2), c-Jun N-terminal kinase 1–3 (JNK 1–3), p38 α, β, γ, δ, and ERK5 cascades, which regulate proliferation, differentiation, and several cellular stresses [2]. The budding yeast *Saccharomyces cerevisiae* possesses five MAPK pathways, which are mediated by the ERK1/2 orthologs Fus3 and Kss1, the p38 ortholog Hog1, the Erk5 ortholog Slt2 (also named Mpk1), and the non-conventional Smk1 MAPK. These MAPK pathways are involved in the control of mating, invasive and pseudohyphal growth, the osmolarity response, the cell wall integrity (CWI), and ascospore formation, respectively [3]. *S. cerevisiae* has been successfully employed as a eukaryotic model, providing very valuable information over the years about the regulation of MAPK cascades and how these pathways propagate signals and modulate cellular responses.

It has long been established that phosphorylation plays a critical role in regulating the timing, duration, and intensity of MAPK signaling. MAPKs share a common posttranslational mechanism of kinase stimulation, characterized by dual phosphorylation of the threonine and tyrosine residues located at their conserved TXY motif within the activation loop. Initial studies showed that MAPKs are only active when both threonyl and tyrosyl residues are phosphorylated [4,5]. Studies performed in mammalian cells revealed that MAPKs are firstly phosphorylated on tyrosine and secondly on threonine by a two-collision distributive mechanism, in which the MAPKK dissociates from the MAPK between both phosphorylation events [6]. Later, a processive manner, meaning that sequential reactions are operated by the same enzyme, was proposed to operate for some mammals and yeast MAPKs [7,8,9,10]. Phosphorylated MAPKs are dephosphorylated in the Thr and/or Tyr residues within the activation loop by protein phosphatases of three different types, including serine–threonine, tyrosine, and Dual-Specificity Phosphatases (DSPs) [11]. Among them, DSPs, also known as MAPK Phosphatases (MKPs), are the main negative regulators [12]. Although it is well-established that MKPs eliminate the phosphate group from both Thr and Tyr residues, the dephosphorylation kinetics have been poorly studied.

Interestingly, reports on several mammalian and yeast MAPKs have highlighted that threonine phosphorylation confers higher catalytical and biological activity than tyrosine phosphorylation [10,13,14,15]. Specifically, the threonine phosphorylation of *S. cerevisiae* Hog1 was found to be essential for its catalytic and biological activity, whereas tyrosine phosphorylation seems to be required to obtain a further increase in activity, only being necessary in extreme conditions [16]. In the case of Kss1, threonine phosphorylation renders the cells capable of inducing invasive growth to some extent [17]. In contrast, the tyrosine monophosphorylated form of Fus3 has been shown to be partially active and competent in the phosphorylation of a variety of substrates in vitro [18]. Moreover, an inhibitory role for the monophosphorylated forms of this yeast MAPK has been reported, indicating that incompletely phosphorylated forms could also act to limit signal transduction [8].

In addition to TXY, MAPK activity is also affected by post-translational modifications (PTMs) in other residues. Thereby, several phosphosites within MAPKs have been experimentally identified, mainly by phosphoproteomic approaches [19,20,21,22]. However, the regulation of most of these phosphorylations, as well as the effect that they have on catalytic and biological activity, remain obscure. In mammalian Erk1, two phosphosites flanking the TEY motif—T207 and Y210—have been reported to negatively regulate its catalytic activity. p-T207 arose from autophosphorylation, whereas the phosphorylation of Y210 was catalyzed by MEK1 [23].

Among the budding yeast MAPK pathways, the CWI pathway is mainly involved in sensing and responding to cell wall stress that arises during morphogenetic processes or under cell wall-threatening conditions. The core module consists of a MAPKK Kinase, Bck1, a pair of redundant MAPK Kinases, Mkk1 and Mkk2, and the MAPK Slt2. Phosphorylation of Slt2 in the TEY motif (T190-E-Y192) makes the MAPK active [24]. Dually phosphorylated Slt2 is mainly located in the nucleus, where it activates transcription factors, among which Rlm1 stands out, and controls the majority of genes that have been shown to be upregulated in response to CWI activation [3,24]. A mutation in Y192, but not in T190, seems to render Slt2 partially functional [25], suggesting the importance of threonine over tyrosine phosphorylation. However, whether Slt2 monophosphorylated forms are present in yeast cells and display a functional role is still unknown.

The aim of this study is to deepen the existing knowledge on the mechanisms regulating the phosphorylation state of the MAPK Slt2. Here, we show the existence and biological relevance of Slt2 monophosphorylated forms, and that both phosphorylation and dephosphorylation of this MAPK are highly ordered, with action on Y192 occurring first. We uncover additional residues within Slt2 that are involved in its activation and biological function.

## 2. Results

### 2.1. Detection of Slt2 Monophosphorylation at the Activation Loop with Commercial Antibodies

**Figure 1:**
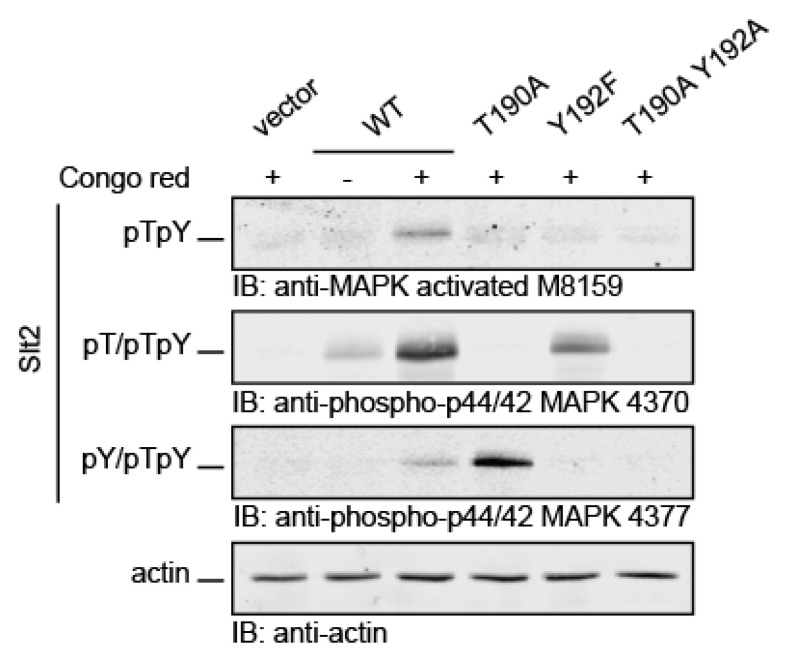
Detection of mono and dually phosphorylated Slt2 species with commercial antibodies. *slt2*Δ (Y00993 strain) cells containing plasmid pRS316 expressing Slt2 (WT), Slt2^T190A^, Slt2^Y192F^, or Slt2^T190A Y192A^ were grown to the mid-exponential phase in yeast extract–bacto-peptone–dextrose (YPD) and then stimulated with 30 µg/mL of Congo Red for 2 h (+). Cell extracts were resolved by SDS–PAGE, and analyzed by immunoblotting with the M8159 antibody (upper panel), which detects dually phosphorylated Mitogen-Activated Protein Kinase (MAPK) (pTpY); the 4370 antibody (middle panel), which detects T190 and dually phosphorylated MAPK (pT/pTpY); and the 4377 antibody (lower panel), which detects Y192 and dually phosphorylated MAPK (pY/pTpY). An anti-actin antibody was used for the loading control. A representative assay from three different experiments with distinct transformants is shown.

The activation status of Slt2 has traditionally been monitored by using commercial antibodies that detect the dually phosphorylated forms of mammalian p42/44 (Erk1/Erk2), which share the same activation motif (TEY) [26]. These antibodies have been used for routinely studying signaling through the CWI pathway, but, according to their specifications, we noticed that some of them could also recognize monophosphorylated forms of MAPKs. For example, the anti-phospho-p44/42 MAPK (Erk1/2) (T202/Y204) (D13.14.4E) XP^®^ rabbit mAb (#4370 Cell Signaling, Danvers, MA, USA) detects endogenous levels of Erk1 and Erk2 when dually phosphorylated and singly phosphorylated at threonine. In turn, the anti-phospho-p44/42 MAPK (Erk1/2) (T202/Y204) (197G2) rabbit mAb (#4377 Cell Signaling) detects Erk1 and Erk2 when dually phosphorylated and individually phosphorylated at tyrosine. In contrast, the anti-MAPK activated (Diphosphorylated ERK-1&2) mouse mAb (#M8159 Sigma-Aldrich, St. Louis, MO, USA) reacts specifically with the dually phosphorylated form and it does not recognize the monophosphorylated species of these MAPKs. Because some monophosphorylated MAPKs have been found to display functional roles, we wanted to explore the actual specificity of these antibodies on Slt2. To this end, extracts from *slt2∆* cells expressing either the dually phosphorylatable wild type Slt2, the monophosphorylatable mutants Slt2^T190A^ and Slt2^Y192F^, or the unphosphorylatable double mutant Slt2^T190A Y192A^, treated with cell wall-altering agent Congo Red (CR) [27], were analyzed by Western blotting using the above-mentioned antibodies. As observed in Figure 1, the three antibodies detected wild type Slt2 and did not recognize unphosphorylatable Slt2^T190A Y192A^. However, #M8159 did not detect any of the monophosphorylatable versions of Slt2, #4370 did not give any signal on Slt2 ^T190A^, and #4377 did not recognize Slt2^Y192F^, indicating that these reagents are only able to detect dual phosphorylation, dual and T190 monophosphorylation, and dual and Y192 monophosphorylation, respectively. The #M8159, #4370, and #4377 antibodies mentioned above are hereafter referred to in this work as anti-Slt2-pTpY, anti-Slt2-pT/pTpY, and anti-Slt2-pY/pTpY, respectively. Interestingly, a comparison of the signals provided by the distinct antibodies on each of the Slt2 versions revealed that, compared with the wild type, the Slt2^T190A^ mutant displayed higher tyrosine monophosphorylation, whereas Slt2^Y192F^ showed lower monophosphothreonine levels. These results indicate that the use of complementary antibodies with distinct phosphosite specificities allowed the study of the different Slt2 phosphoforms.

### 2.2. The Lack of T190 Phosphorylation Results in Increased Phosphorylation of Y192 in Slt2

**Figure 2:**
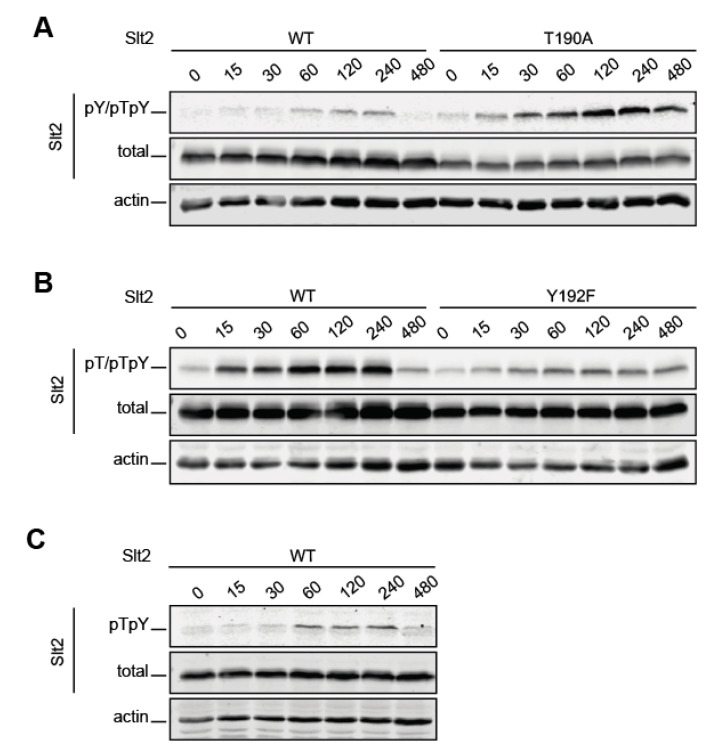
Analysis of Slt2 phosphorylation dynamics. BY4741 *slt2Δ*-RLM1Myc cells containing the plasmid pRS316 expressing, as indicated, Slt2 (WT), Slt2^T190A^, or Slt2^Y192F^, were grown to the mid-exponential phase in YPD and then stimulated with 30 µg/mL of Congo Red for the indicated times (min). Cell extracts were resolved by SDS–PAGE, and analyzed by immunoblotting with anti-Slt2-pY/pTpY (**A**), anti-Slt2-pT/pTpY (**B**), and anti-Slt2-pTpY (**C**), as in Figure 1; anti-Mpk1, which detects the total Slt2; and anti-actin antibodies (**A**–**C**). A representative assay from three different experiments with distinct transformants is shown.

To deepen the characterization of dual phosphorylation dynamics at the TEY motif of Slt2, time-course monitoring of Slt2 T190 and Y192 phosphorylation following Congo Red treatment of yeast cells was performed. As observed in Figure 2, the wild type Slt2 signal provided by the three antibodies increased over time, reaching a plateau between 60 and 120 min, which remained until 240 min, when it then decreased to basal levels at 480 min. Although the individual phosphosite Slt2 mutants displayed similar phosphorylation dynamics to the wild type form, several differences were also visible. Changing T190 to alanine resulted in an increase in Y192 phosphorylation (Figure 2A), whereas a lack of Y192 led to a diminished phosphorylation of T190 (Figure 2B) when compared to wild type Slt2 phosphorylation levels. Moreover, both Slt2 mutant versions exhibited a less pronounced decrease in the phosphorylation signal after the plateau than the wild type protein, without reaching basal levels at 480 min. Altogether, these results suggest that threonine and tyrosine residues have a fundamental and likely opposite role in the maintenance of the MAPK phosphorylation status. The tyrosine is needed for Slt2 to reach its wild type dual phosphorylation level, while the threonine residue is somehow involved in the negative regulation of Slt2 tyrosine phosphorylation.

### 2.3. Y192 Phosphorylation by Mkk1 and Mkk2 Is a Stepping-Stone to the Dual Phosphorylation of Slt2

**Figure 3:**
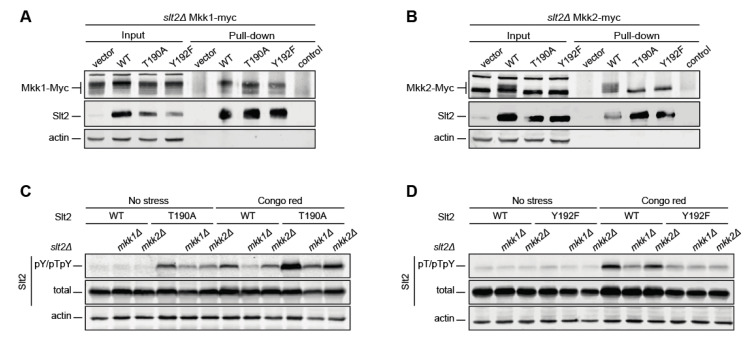
Analysis of Mkk1 and Mkk2 binding to differentially phosphorylatable forms of Slt2 and their effect on Slt2 phosphorylation. The strains *slt2*Δ *MKK1-myc* (YMJ30) (**A**) and *slt2*Δ *MKK2-myc* (YMJ31) (**B**) were transformed with the plasmid pRS316 alone (vector) or expressing, as indicated, Slt2 (WT), Slt2^T190A^, or Slt2^Y192F^. Cells were grown to the mid-exponential phase in YPD and then stimulated with 30 µg/mL of Congo Red (CR) for 2 h. Cell extracts (input) were incubated with the Mpk1 antibody attached to Dynabeads™ Protein G to purify Slt2 complexes (pull-down) and resolved by SDS–PAGE. Proteins were detected with anti-c-Myc, anti-Mpk1 (Slt2), and anti-actin antibodies by immunoblotting. The control of a Dynabeads-Mpk1 antibody non-incubated with cell extracts is included. (**C**,**D**) *slt2*Δ (Y00993 strain), *mkk1*Δ *slt2Δ* (YGGR32 strain), and *mkk2*Δ *slt2*Δ (YGGR33 strain) cells containing the same plasmids as in (A,B) were grown to the mid-exponential phase in YPD and then stimulated with 30 µg/mL of CR for 2 h. Cell extracts were resolved by SDS–PAGE, and analyzed by immunoblotting with anti-Slt2-pY/pTpY, anti-Slt2-pT/pTpY (as in Figure 1), anti-Mpk1 (total), and anti-actin antibodies. A representative assay from three different experiments with distinct transformants is shown.

The increased Y192 phosphorylation in the absence of T190 could be due to an intensification of Slt2^T190A^’s interaction with its activating MAPKKs, Mkk1, and/or Mkk2. Similarly, the slight reduction in T190 phosphorylation in the Y192F version could be because of a diminished interaction with the activator kinases. To check this, we carried out copurification experiments of wild type, T190A, and Y192F versions of Slt2 with Mkk1-myc and Mkk2-myc expressed from genomic-tagged *MKK1* and *MKK2*, respectively. As observed in Figure 3A,B, neither Mkk1 nor Mkk2 showed higher retention by Slt2^T190A^ when compared to Slt2, indicating that the increased Y192 phosphorylation of this mutant protein was not caused by a trapping effect by either Mkk1 or Mkk2. Similarly, Slt2^Y192F^ bound the same amount of Mkk1 and Mkk2 as Slt2^T190A^ and Slt2, revealing that these mutations do not result in differences in binding to their activators.

We have shown that Mkk1 plays a preeminent role over Mkk2 in signaling through the CWI pathway [28]. To assess whether these Mkks act differentially on T190 and Y192 Slt2 phosphosites, we analyzed the impact of eliminating individual MAPKKs on monophosphorylatable Slt2 mutant versions. *MKK1* deletion severely reduced the CR-induced Y192 phosphorylation of both wild type and T190A Slt2 versions. The lack of Mkk2 also resulted in a similar, although less intense, decrease (Figure 3C). The same effect was observed for the T190 phosphorylation of wild type Slt2 when any of these MAPKKs was eliminated. In contrast, the lack of either Mkk1 or Mkk2 did not lead to any change in the T190 phosphorylation of Slt2 ^Y192F^ (Figure 3D, lanes 10–12). These findings suggest that priming phosphorylation at Y192 is mainly carried out by Mkk1, and that this modification is necessary for subsequent phosphorylation at T190.

### 2.4. Dephosphorylation of T190 on Slt2 by Msg5 Depends on the Previous Dephosphorylation of Y192

**Figure 4:**
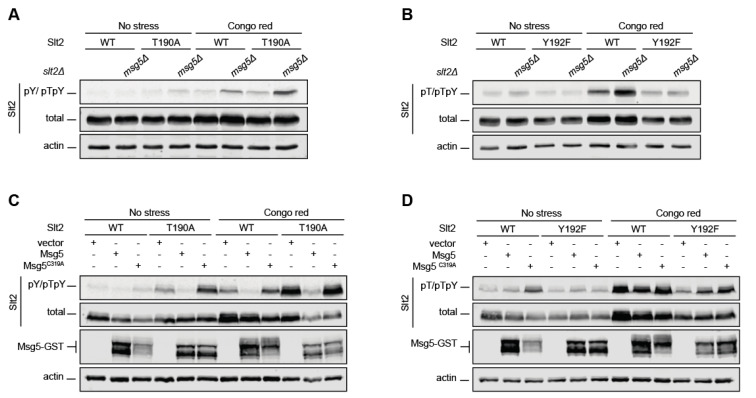
Analysis of the dephosphorylation and trapping of differentially phosphorylatable forms of Slt2 by Msg5. (**A**,**B**) *msg5*Δ *slt2*Δ (YGGR34 strain) cells containing the plasmid pRS316 expressing, as indicated, Slt2 (WT), Slt2^T190A^, or Slt2^Y192F^, were grown to the mid-exponential phase in YPD and then stimulated with 30 µg/mL of CR for 2 h. Cell extracts were resolved by SDS–PAGE, and analyzed by immunoblotting with anti-Slt2-pY/pTpY and anti-Slt2-pT/pTpY, as in Figure 1; anti-Mpk1 (total); and anti-actin antibodies. (**C**,**D**) Yeast extracts from *slt2*Δ (Y0093 strain) cells co-transformed with the plasmid pRS315 bearing the same Slt2 versions as in (**A**,**B**), and with YEp352 expressing Msg5 or Msg5^C319A^ fused to Glutathione S-transferase (GST), were resolved by SDS–PAGE and analyzed by immunoblotting with the same antibodies as in (**A**,**B**), as well as with an anti-GST antibody. A representative assay from three different experiments with distinct transformants is shown.

Slt2 phosphorylation is regulated by different protein phosphatases, including the Dual-Specificity Phosphatase (DSP) Msg5 [11,12]. Since DSPs dephosphorylate both tyrosine and threonine residues, we reasoned that the increased Y192 phosphorylation observed in Slt2^T190A^ could be due to the reduced activity of Msg5 on phosphorylated tyrosine in the absence of phosphorylated threonine. However, the lack of Msg5 in cell wall-stressed cells resulted in increased Y192 phosphorylation of both Slt2 and Slt2^T190A^ (Figure 4A), whereas no effect was observed on the T190 phosphorylation of Slt2^Y192F^ (Figure 4B). This indicates that Msg5 can act on Y192-monophosphorylated Slt2, but not on T190-monophosphorylated Slt2.

We have previously shown that Msg5 overexpression leads to Slt2 dephosphorylation [29], while the overexpression of catalytically inactive Msg5^C319A^ results in an increase in the amount of phosphorylated Slt2 due to a trapping effect [30]. As observed in Figure 4C, Msg5 overexpression reduced the phosphorylation level of Y192 in both Slt2 and Slt2^T190A^, and Msg5^C319A^ overexpression led to an increase in Y192 phosphorylation in both species, indicating the ability of Msg5 and Msg5^C319A^ to dephosphorylate and trap Y192-phosphorylated Slt2, respectively, independent of T190 phosphorylation. In contrast, no changes in the phosphorylation status of T190 in Slt2^Y192F^ following the overexpression of either Msg5 or Msg5^C319A^ were observed (Figure 4D). Consequently, neither Msg5 nor Msg5^C319A^ can dephosphorylate or trap T190-phosphorylated Slt2, respectively, in the absence of phosphorylated Y192. This indicates that the dephosphorylation of tyrosine is needed for effective threonine dephosphorylation at the activation loop of Slt2 and reinforces the idea that the Slt2 threonyl-phosphorylated residue is a poor substrate for this DSP.

Taken together, these findings suggest that, despite being able to dephosphorylate both threonine and tyrosine residues within the TEY motif, the DSP Msg5 carries out an ordered dephosphorylation of Slt2, which is initiated at Y192.

### 2.5. T190 Is Essential for the Catalytic Activity and Biological Role of Slt2

**Figure 5:**
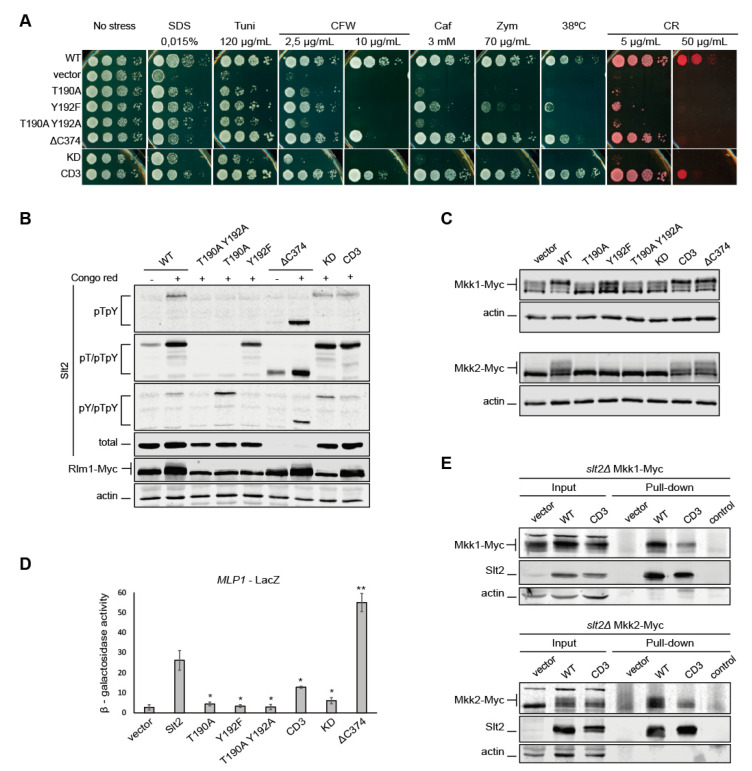
Analysis of the activity of different mutant versions of Slt2. (**A**) Ten-fold serial dilutions of BY4741 *slt2*Δ-RLM1Myc cells bearing the pRS316 plasmid alone (vector) or expressing Slt2 (WT), Slt2^T190A^, Slt2^Y192F^, Slt2^T190A Y192A^, Slt2^ΔC374^, Slt2^KD^, or Slt2^CD3^. Cells were spotted onto YPD plates in the absence (no stress) or presence of Sodium Dodecyl Sulfate (SDS), tunicamycin (Tuni), Calcofluor White (CFW), caffeine (Caf), zymolyase (Zym), and Congo Red (CR) at the indicated concentrations. (**B**) The same cells as in (**A**) were grown to the mid-exponential phase in YPD and then stimulated with 30 µg/mL of CR for 2 h. Cell extracts were resolved by SDS–PAGE, and analyzed by immunoblotting with anti-Slt2-pTpY, anti-Slt2-pT/pTpY, and anti-Slt2-pY/pTpY, as in Figure 1; anti-Mpk1 (total); anti-Myc 4A6; and anti-actin antibodies. (**C**) Extracts from *slt2*Δ *MKK1-myc* (YMJ30 strain) or *slt2*Δ *MKK2-myc* (YMJ31 strain) cells transformed with the same plasmids as in (**A**) were resolved by SDS–PAGE, as in (**B**), and analyzed by immunoblotting with anti-Myc 4A6 and anti-actin antibodies. (**D**) β-galactosidase activity of cell extracts from *slt2*Δ (Y00993) cells co-transformed with p*MLP1-LacZ* and the same plasmids as in (**A**) after incubation with 30 µg/mL of CR for 2 h. Data represent the average of the activity of three independent transformants. Error bars indicate the standard deviation. Asterisks (* and **) indicate *p*-values of <0.05 and <0.01, respectively, assessed by Student’s t-test, and refer to the Slt2 wild type (WT) activity. (**E**) The same strains as in (C), transformed with the pRS316 plasmid expressing Slt2 (WT) or Slt2^CD3^. Cells were grown to the mid-exponential phase in YPD and stimulated with 30 µg/mL of CR for 2 h. Cell extracts (input) were incubated with the anti-Mpk1 antibody attached to Dynabeads™ Protein G to purify Slt2 complexes (pull-down) and resolved by SDS–PAGE. Proteins were detected with anti-c-Myc, anti-Mpk1 (Slt2), and anti-actin antibodies by immunoblotting. A control of the Dynabeads-Mpk1 antibody non-incubated with cell extracts is included. A representative assay from three different experiments with distinct transformants is shown.

Initial work by Lee et al. in 1993 [25] showed that the mutation of threonine 190 to alanine (T190A) resulted in an *SLT2* allele that was incapable of complementing the thermosensitivity of an *SLT2*-deleted strain. In contrast, the mutation of tyrosine 192 to phenylalanine (Y192F) resulted in a partially functional allele that poorly complemented this phenotype. To further ascertain the biological relevance of each of these phosphorylation sites, we first analyzed the ability of these mutants to complement the sensitivity of *slt2*∆ cells to distinct stresses that trigger activation of the CWI pathway [31] in drop growth assays. As shown in Figure 5A, *slt2Y192F* showed partial complementation of the sensitivity of *slt2*∆ cells not only to 38 °C, but also to caffeine, zymolyase, sodium dodecyl sulfate (SDS), tunicamycin, and low concentrations of Calcofluor White (CFW) and CR, which indicates that T190-monophosphorylated Slt2 retains some activity in the absence of Y192 phosphorylation. In contrast, both *slt2T190A* and the non-phosphorylatable *slt2T190A Y192A* behaved like the kinase-dead version *slt2K54F* (*slt2*KD) [32], not allowing growth of *slt2*∆ cells in most of the assayed conditions, and thus evidencing the loss of activity caused by these mutations. Curiously, transformants bearing any of these inactive alleles were able to grow slightly more than *slt2*∆ cells carrying the empty vector in the presence of SDS, tunicamycin, caffeine, and a low CFW concentration. These results suggest that inactive Slt2 could still play a biological role by mediating a response that is strong enough to partially cope with these stresses through a mechanism independent of the phosphorylation of the TEY motif within its activation loop.

We also tested other readouts of Slt2 activity on the phosphosite mutant versions, namely, the in vivo competence to (i) phosphorylate and consequently promote an SDS–PAGE shift in the transcription factor Rlm1 [33] (Figure 5B) and the MAPKKs Mkk1 and Mkk2 [34] (Figure 5C), (ii) increase its own protein level [35] (Figure 5B), and (iii) transcriptionally induce the Rlm1-dependent *MLP1* promoter by using a reporter transcriptional assay with *lacZ* [36] (Figure 5D). Only Slt2^Y192F^ showed any activity in one of the analyzed molecular readouts, as revealed by the partial mobility upshift observed for Mkk1-myc (Figure 5C).

We next extended these activity studies to additional Slt2 mutant versions affected at relevant sites or domains that could lead to changes in their phosphorylation status. MAPKs bind activating kinases and other interacting proteins such as phosphatases or transcription factors via a major docking site named “Common Docking” (CD). An Slt2 mutant version carrying substitutions of the conserved acidic residues D326, D323, and E327 for asparagine within the CD of Slt2 (Slt2^CD3^) [37] was analyzed. Slt2^CD3^-expressing cells displayed a wild type phenotype, except when exposed to high concentrations of CR and CFW, in which their ability to grow was slightly reduced. T190 and Y192 phosphorylation of Slt2^CD3^ was similar to that of wild type Slt2 (Figure 5B). Copurification experiments indicated that Slt2^CD3^ was able to bind to both Mkk1 and Mkk2, although the amount of these proteins pulled down by wild type Slt2 was much higher (Figure 5E). Furthermore, Rlm1 phosphorylation (Figure 5B) and *MLP1*-*LacZ-*driven β-galactosidase levels (Figure 5D) were also decreased in Slt2^CD3^-expressing cells. All these results suggest that there must be other interaction sites in Slt2, in addition to the CD domain, to bind Mkk1 and Mkk2 and probably Rlm1.

Removal of the C-terminal region of MAPKs Slt2 and Hog1 has been proven to render them spontaneously and highly phosphorylated [38]. To analyze this phenomenon in more detail, we constructed an Slt2 version lacking their 111 C-terminal amino acids (Slt2^ΔC374^). As observed in Figure 5B, Slt2^ΔC374^ displayed higher phosphorylation than Slt2 under stress. However, in basal conditions, the C-terminal truncated form only showed higher phosphorylation than wild type Slt2 on T190, and not on Y192 (Figure 5B). According to the increased phosphorylation, Slt2^ΔC374^ displayed higher activity than wild type Slt2, as determined by the *MLP1*-*LacZ* expression (Figure 5D). This could affect the functionality of Slt2^ΔC374^ in vivo, since this truncated kinase did not totally restore growth of the *slt2*∆ strain under CR and CFW at high concentrations and SDS treatment (Figure 5A).

### 2.6. T195 Plays a Key Role in Slt2 Activity

**Figure 6:**
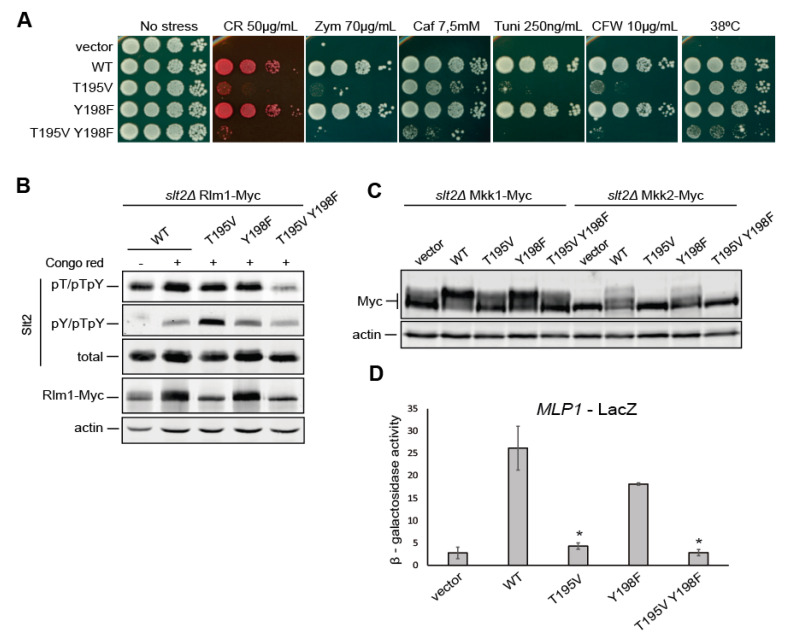
Analysis of the activity of Slt2 mutated in the conserved residues T195 and Y198. (**A**) Ten-fold serial dilutions of BY4741 *slt2*Δ-RLM1Myc cells bearing the pRS316 plasmid alone (vector) or expressing Slt2 (WT), Slt2^T195V^, Slt2^Y198F^, or Slt2^T195V Y198F^. Cells were spotted onto YPD plates in the absence (no stress) or presence of the indicated stimuli. (**B**) The same cells as in (**A**) were grown to the mid-exponential phase in YPD and stimulated with 30 µg/mL of CR for 2 h. Cell extracts were resolved by SDS–PAGE, and analyzed by immunoblotting with anti-Slt2-pT/pTpY and anti-Slt2-pY/pTpY, as in Figure 1; anti-Mpk1 (total); anti-MYC 4A6; and anti-actin antibodies. (**C**) Yeast extracts from *slt2*Δ *MKK1-myc* (YMJ30 strain) and *slt2*Δ *MKK2-myc* (YMJ31 strain) cells transformed with the same plasmids as in (**A**) were resolved by SDS–PAGE as in (**B**) and analyzed by immunoblotting with anti-Myc 4A6 and anti-actin antibodies. (**D**) β-galactosidase activity of cell extracts from *slt2Δ* (Y00993 strain) co-transformed with p*MLP1-LacZ* and the same plasmids as in (**A**) after incubation with 30 µg/mL of CR for 2 h. Data represent the average of the activity of three independent transformants. Error bars indicate the standard deviation. One asterisk (*) indicates a *p*-value of <0.05, assessed by Student’s t-test, and refers to wild type Slt2 (WT) activity. A representative assay from three different experiments with distinct transformants is shown.

The phosphorylation of two Erk1 phosphosites—T207 and Y210—flanking the TEY motif, has been reported to negatively regulate the catalytic activity of this MAPK [23]. Since these two residues are conserved in Slt2, we sought to evaluate the functional relevance of their Slt2 counterparts T195 and Y198. To this end, Slt2 mutants affected at these sites were created by substitution of the T195 residue with valine and the Y198 residue with phenylalanine. Drop growth assays revealed that whereas Slt2^Y198F^ fully complemented the sensitivity to typical CWI pathway stress agents of an *slt2*∆ mutant, Slt2^T195V^ and Slt2^T195V Y198F^ did not restore cell growth in the presence of tunicamycin, zymolyase, CR, and CFW. However, Slt2^T195V^ provided resistance to *slt2*∆ cells under high temperature or caffeine stress, suggesting some functionality for this mutant protein. Interestingly, the substitution Y198F displayed a synthetic effect with T195V under all these stresses (Figure 6A). Rlm1-Myc, Mkk1-Myc, and Mkk2-Myc mobility shift assays revealed that Slt2 versions bearing the T195V mutation, either individually or combined with Y198F, were not able to phosphorylate any of these Slt2 substrates. In contrast, Slt2^Y198F^ clearly promoted their characteristic mobility upshift (Figure 6B,C). In agreement, both Slt2^T195V^ and Slt2^T195V Y198F^, but not Slt2^Y198F^, were unable to induce Rlm1-dependent *MLP1* transcription (Figure 6D). All these results support the conclusion that T195V mutation severely reduces Slt2 activity and, together with Y198F mutation, abrogates the Slt2 function. The autophosphorylation of Erk2 at T188 (equivalent to T195 in Slt2) was reported to promote its nuclear localization [39], and Y210F mutation (equivalent to Y198F in Slt2) was recently found to drastically inhibit Erk1 nuclear translocation [40]. Therefore, we also evaluated the possibility of T195 or Y198 playing a role in Slt2 localization by constructing N-terminally green fluorescent protein (GFP)-tagged versions of Slt2, Slt2^T195V^, and Slt2^T195V Y198F^. Fluorescent microscopy analysis determined that both GFP-Slt2^T195V^ and GFP-Slt2^T195V Y198F^ showed the same localization pattern as GFP-Slt2, displaying predominantly nuclear localization, either at basal conditions or under heat treatment (Supplementary Figure S1).

### 2.7. T190 and T195 Are Essential for Maintaining a Low Level of Y192 Phosphorylation

**Figure 7:**
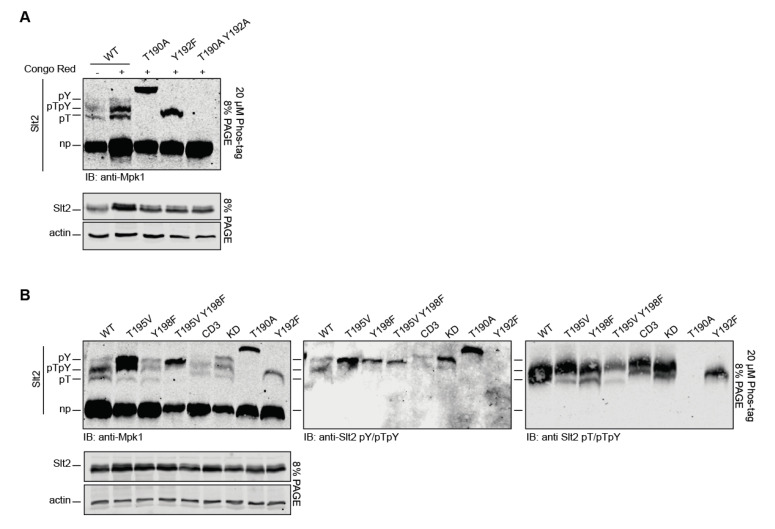
Detection by phosphate-affinity (Phos-tag) of the differentially phosphorylated forms of Slt2. (**A**) BY4741 *slt2Δ*-RLM1Myc cells containing the same plasmid pRS316 expressing Slt2 (WT), Slt2^T190A^, Slt2^Y192F^, or Slt2^T190A Y192A^ were grown to the mid-exponential phase in YPD and then stimulated with 30 µg/mL of CR for 2 h. Cell extracts were resolved by Phos-tag SDS–PAGE (top panel) and SDS–PAGE (bottom panel), and immunoblotted with anti-Mpk1 (Slt2) and anti-actin antibodies. (**B**) Cell extracts of *slt2*Δ-RLM1Myc cells containing plasmid pRS316 expressing the same proteins as in (**A**), as well as Slt2^T195V^, Slt2^Y198F^, Slt2^T195V Y198F^, Slt2^CD3^, and Slt2^KD^, resolved as in (**A**) and immunoblotted with anti-Mpk1 (Slt2) and anti-actin (left panels), anti-Slt2-pY/pTpY (middle panel), and anti-Slt2-pT/pTpY (right panel) antibodies. A representative assay from three different experiments with distinct transformants is shown.

The use of antibodies with different specificities for each phosphorylated Slt2 form has proved useful for tracking the phosphorylation of the individual phosphosite mutants analyzed in this work. Nevertheless, each antibody has a different sensitivity, so the signals are not comparative. In order to monitor the abundance of each of the phosphorylation states of wild type Slt2, we used phosphate-affinity SDS–PAGE (Phos-tag SDS–PAGE). Phos-tag is a tool based in a functional molecule, which, in the presence of Zn2+, forms complexes with phosphorylated serine, threonine, and tyrosine, inducing mobility shifts in phosphorylated proteins, depending on their phosphorylation status [41]. When Slt2 was analyzed by this method, three upshifting bands were observed after immunoblotting with an antibody that recognizes total Slt2, indicating the existence of three different phosphorylation species, in addition to the most abundant and fastest migrating band corresponding to non-phosphorylated Slt2 (Figure 7A). The stimulation of cells with CR led to an increase in the intensity of these three bands, but they still represented a very low proportion of the whole pool of Slt2. Individual phosphosite mutants Slt2^T190A^ and Slt2^Y192F^ yielded just one upshifting band with different mobility patterns, corresponding to monophosphorylated species. Accordingly, none of the phosphorylated species were detected in the double Slt2^T190A Y192A^ mutant. As shown in Figure 7B, immunodetection with anti-Slt2-pY/pTpY and anti-Slt2-pT/pTpY antibodies revealed that, among the three upshifting species, the slowest and least intense band corresponded to Slt2 monophosphorylated at Y192; the middle and most intense band to dually phosphorylated Slt2; and the fastest, with an intermediate level of intensity, to T190-monophosphorylated Slt2. Strikingly, the Y192-monophosphorylated form of the Slt2^T190A^ mutant migrated more slowly than that of wild type Slt2. Remarkably, the analysis by Phos-tag SDS–PAGE confirmed the strong increase in the amount of the Y192-monophosphorylated form caused by the lack of T190 observed by conventional SDS–PAGE (Figure 1 and Figure 2A).

The T195V mutation promoted a strong accumulation of the Y192-monophosphorylated Slt2 form, and a reduction in T190 monophosphorylation (Figure 7B, left panel). The Y198F mutation yielded a similar decrease in T190-monophosphorylated Slt2, but only a slight increase in Y192-monophosphorylated Slt2. The combination of both mutations diminished the hyperphosphorylation at Y192 caused by T195V mutation and further reduced monophosphorylation at T190 to almost negligible levels. A strong reduction in the dually phosphorylated form was also observed for the double mutant Slt2^T195V Y198F^. Immunodetection with anti-Slt2-pY/pTpY (Figure 7B, middle panel) and anti-Slt2-pT/pTpY (Figure 7B, right panel) of the same extracts confirmed the clogging of the Y192-monophosphorylated form of Slt2^T195V^ and the strong reduction in T190-monophosphorylation and dual phosphorylation of Slt2^T195V Y198F^; effects that were also observed by conventional SDS–PAGE (Figure 6B).

An analysis of Slt2^KD^ and Slt2^CD3^ by the Phos-tag method revealed the same three phosphorylated bands, but with a slightly lower mobility than those of the wild type Slt2 (Figure 7B). Interestingly, the Slt2^KD^ mutant lacking kinase activity displayed an increased level of Y192 monophosphorylation, whereas Slt2^CD3^ showed an overall decrease in the amount of all phosphorylated forms, in agreement with the reduced binding to the activating kinases Mkk1 and Mkk2.

## 3. Discussion

By combining the use of phosphosite-specific antibodies with the Phos-tag methodology in Western blotting analysis, we have taken a step forward in the characterization of signaling through the CWI pathway at the MAPK Slt2 level. In addition to setting up methodological tools for monitoring the abundance of the distinct co-existing Slt2 phosphoforms, we have deepened the knowledge on this yeast MAPK in terms of (i) the impact of individual phosphorylation events on functionality, (ii) the order of phosphorylation and dephosphorylation reactions within the TEY motif, (iii) the Slt2 phosphorylation requirements for key activators and down regulators to act on this MAPK, and (iv) the molecular and functional relevance of several key motifs of Slt2.

Activation of the CWI pathway has long been known to correlate with an increased phosphorylation of Slt2 [26]. Therefore, to evaluate the activity of this MAPK, ours and other research groups studying fungal CWI pathways have traditionally used commercial antibodies that detect the dually phosphorylated species of mammalian ERKs [42]. However, some of these antibodies are also able to detect ERK monophosphorylated forms, which have been shown to display disparate signaling roles. Although in vitro analysis of Erk2 revealed that the two monophosphorylated forms produced an intermediate activity state of this MAPK [43], most studies in mammalian MAPKs have reported that only the monophosphothreonyl residue displayed some biological activity [13,15]. This is also the case for Pmk1, which is the Slt2 homolog in the fission yeast *Schizosaccharomyces pombe* [10]. Here, we found that threonine-monophosphorylated Slt2^Y192F^ is partially active, whereas Slt2^T190A^ only retained very residual activity, similar to that of catalytically inactive Slt2^KD^, suggesting that a non-catalytic mechanism underlies the limited effect of the Y192-monophosphorylated form. Furthermore, we have shown that, in contrast to Slt2, the threonine monosphosphorylatable Slt2^Y192F^ mutant was able to retrophosphorylate Mkk1, but not Mkk2, suggesting that the different phosphoforms may have distinct substrate specificities, as observed for p38α [44]. Intriguingly, by using the antibody that recognizes monophosphorylation at Y192, as well as Phos-tag analysis, we observed that changes in specific Slt2 residues that render an activity-deficient MAPK, such as Slt2^T190A^, Slt2^KD^, and Slt2^T195V^, correlate with hyperphosphorylation of the Y192. This demonstrates that an increase in the phosphorylation signal does not always correlate with an increase in the activity of the MAPK and shows the importance of discerning the different phosphorylation states of an MAPK by using complementary antibodies, and ideally, the Phos-tag methodology.

In this study, we characterized, for the first time, the Slt2 phosphorylation state by using Phos-tag analysis. In other yeast MAPKs, such as Hog1 and Kss1, only dual-phosphorylated forms have been detected [9,45]. In the case of Fus3, two upshifting bands—one corresponding to monophosphorylated and the other to dual-phosphorylated forms—were observed [8]. However, here, we have shown that three upshifting bands, corresponding to threonine-monophosphorylated, tyrosine-monophosphorylated, and dually-phosphorylated Slt2, can be detected by using Phos-tag. Strikingly, even upon stimulation, phosphorylated Slt2 levels are very low relative to the whole pool of Slt2 present in the yeast cell. In contrast, in Fus3 and Kss1, the total amount of phosphorylated forms is quite similar to the non-phosphorylated MAPKs [8,45], while the entire pool of cellular Hog1 is dually phosphorylated in response to osmotic stress [9]. Although the role that this high level of unphosphorylated Slt2 plays in shaping the response of this pathway to cell wall stresses remains to be established, this finding suggests that activated Slt2 is likely to exist only in discrete pools at specific cellular localizations or compartments. Future studies on Slt2 phosphorylation triggered by different CWI stimuli [31] using Phos-tag analysis would be very useful for identifying stress conditions that render higher levels of phosphorylated Slt2.

Here, we have shown that, following stress, when phosphorylation on Y192 is constrained, T190 is rarely phosphorylated and, in clear contrast, when the phosphorylation of T190 is inhibited, Y192 phosphorylation accumulates, leading to the “clogging” of Y192-monophosphorylated Slt2. Similar behavior has also been described for *S. pombe* Pmk1 [10]. Our results prove that this hyperphosphorylation on the Y192 residue is neither caused by a trapping mechanism of the Slt2^T190A^ mutant version by any of the MKKs, nor by a lack of Msg5 phosphatase activity on the Y192 residue. On the contrary, Msg5 preferentially dephosphorylates the phosphotyrosine residue. Together, these results suggest that dual phosphorylation and dephosphorylation is a highly ordered mechanism in which Y192 phosphorylation can be considered as a stepping-stone for full Slt2 activation/inactivation, while T190 phosphorylation is responsible for providing activity to the MAPK. The same phosphorylation and desphosphorylation order was described in the mammalian Erk1 [46,47,48]. Although initial studies indicated that the dual phosphorylation of MAPKs by MAPK kinases occurred in a two-step, distributive manner [49], a later study showed that mammalian MAPK Erk could be phosphorylated in a processive manner due to molecular crowding [7]. This was as also suggested for Pmk1 and Sty1 in the rod-shaped yeast *S. pombe* [10], and for Fus3 and Hog1 in *S. cerevisiae* [8,9]. Considering the lower level of tyrosine-monophosphorylation and dual phosphorylation compared to unphosphorylated Slt2, and the similar phosphorylation dynamics monitored with the different antibodies, Slt2 phosphorylation by Mkk1 and Mkk2 seems to occur via a processive mechanism.

Our results also allowed us to gain an insight into the impact of residues and domains of Slt2 other than TEY on its activation by phosphorylation and/or activity, reflecting the complexity of its regulation. Although it has been described that the interaction of the Erk CD domain is crucial for binding to D domains of MKPs [50], in previous studies, we demonstrated that, rather than mediating the interaction with Msg5, the CD domain was involved in Slt2 binding to Mkk1 [37]. Here, we have shown that both Mkks bind Slt2 not only through the CD site, but also in a multivalent way, as occurs with the MEK–Erk interaction [51,52]. Similarly, although D domain mutations in Rlm1 blocked transcriptional activation [53], we found that the Slt2^CD3^ mutant protein is still able to signal to Rlm1, suggesting that alternative sites also exist in Slt2 for Rlm1 D domain interaction. Whereas the lack of a functional CD domain reduced Slt2 activity, removal of the C-terminal part of Slt2 rendered it hyperactive. A similar truncated Slt2 version was previously observed to be hyperphosphorylated by using an anti-phospho-ERK antibody that detects Tyr 204 phosphorylation within the TEY motif [38]. These authors suggested that Slt2 has an inherent autophosphorylation capability that is suppressed via the C-terminal domain. We observed that Slt2^ΔC374^ also displays hyperphosphorylation at the T190 residue, supporting the idea that the Slt2 tail negatively regulates autophosphorylation on T190 and, ultimately, the activity of the kinase.

In most MAPKs, modifications other than TEY phosphorylation at the activation loop are essential for regulating kinase activity. T195 and Y198 of Slt2 are equivalent positions to phosphosites T207 and Y210 in mammalian Erk1, which are two highly conserved amino acids found not only in MAPKs, but also in most human protein kinases [23]. However, in our Phos-tag analysis, no Slt2 phosphorylation in addition to that found at the TEY motif was detected, at least under CR stress. Whereas Y210 in Erk1 is essential for its recognition by MEK1, we found that the Y198F mutation only had a minor effect on Slt2 phosphorylation and activity, suggesting that Y198 is not an essential regulatory site in Slt2. In contrast, T195 seems to be important for Slt2 activity and functionality, as reported for Erk1, in which T207A mutation resulted in a reduction in its kinase activity [23]. Slt2^T195V^ is highly phosphorylated on Y192, as mentioned above for non-active Slt2 proteins, such as Slt2^T190A^ or Slt2^KD^. This phenomenon could be due to a compensatory increase in the activity of upstream elements along the route, caused by cell wall defects provoked by the absence of a functional CWI pathway. Additionally, inactive Slt2 mutants would be unable to trigger negative feedback loops, such as retrophosphorylation of Mkk1 and Mkk2 [34].

MAPKs from yeast to humans have the TXY motif in common, whose dual phosphorylation is required for them to be fully active. Nevertheless, results such as those presented in this study show that monophosphorylated forms can play different biological roles and that conserved residues other than those in the TEY motif influence the activity, even having opposite effects among MAPKs. This underscores the importance of deepening the study of each MAPK in order to provide a broad view of the complexity of this family of kinases, which, although highly conserved, also shows a great variety of regulatory mechanisms.

## 4. Materials and Methods

### 4.1. Plasmids and DNA Manipulation

Plasmids used in this study (Table 1) were generated using standard DNA techniques, with *Escherichia coli* strain DH5α as the host. All employed oligonucleotides are listed in Supplementary Table S1. The fidelity of all constructs was verified by nucleotide sequence analysis.

To obtain pRS316-*slt2*^T190A^ and pRS316-*slt2*^Y192F^, the 1.3 kb *Hind*III–*Hind*III fragments from YCp50-*slt2*^T190A^, and YCp50- *slt2*^Y192F^ plasmids were cloned into the 5.8 kb *Hind*III–*Hind*III fragment of pRS316-*SLT2*. The pRS315-*SLT2*, pRS315-*slt2*^T190A^, and pRS315-*slt2*^Y192F^ plasmids were constructed by inserting the 2.8 kb *Sac*I-*BamH*I fragment from pRS316-*SLT2*, pRS316-*slt2*^T190A^, or pRS316-*slt2*^Y192F^ into the pRS315 vector cleaved with the same endonucleases.

An overlapping PCR with *SLT2*-5 and *SLT2*-3 primers, in combination with mutagenic primers *SLT2*-MNP5/*SLT2*-MNP3, *SLT2*-MSTOP5/*SLT2*-MSTOP3, and *SLT2*-CD5/*SLT2*-CD3, was carried out to construct pRS316-*slt2*^T190A Y192A^, pRS316-*slt2*^ΔC374^, and pRS316-*slt2*^CD3^, respectively, by using pRS316-*SLT2* as the template. The *Hind*III–*Hind*III digested PCR products were inserted into the 5.8 kb *Hind*III–*Hind*III fragment from pRS316-*SLT2*. The kinase-dead version of *SLT2* (pRS316-*slt2*^KD^) was obtained by subcloning the *EcoR*I–*Sal*I 2.2-kb fragment bearing *slt2-K54F* from pHR3 into *EcoR*I–*Sal*I sites of pRS316.

pRS316-*slt2*^T195V^, pRS316-*slt2*^Y198F^, and pRS316-*slt2*^T195V Y198F^ were obtained by site-directed mutagenesis with primers *SLT2*-T195V5/*SLT2*-T195V3, *SLT2*-Y198F5/*SLT2*-Y198F3, and *SLT2*-T195VY198F5/*SLT2*-T195VY198F3, respectively, using the plasmid pRS316-*SLT2* as the template, and followed by *Dpn*I digestion.

The plasmid pRS316-*GFP*-*SLT2* was constructed via a two-step process. First, the fragment *Cla*I-6xHis-*Mlu*I was introduced into the N-terminal region of *SLT2* by an overlapping PCR with primers *SLT2*-Pre*Sal*I.5, *SLT2*-Post*EcoR*I.3, *SLT2*-*ClaI*6×His*MluI*.5, and *SLT2*-*ClaI*6×His*MluI*.3, using pRS316-*SLT2* as the template to generate pRS316-6×His-*SLT2.* Second, *GFP* was amplified from the pLA10 plasmid with primers *GFP*-5 and *GFP*-3, which include *Cla*I and *Mlu*I restriction sites, respectively. The PCR product was then digested and subcloned into *Cla*I–*Mlu*I sites of pRS316-6xHis-*SLT2,* producing pRS316-*GFP*-*SLT2.* Plasmids pRS316-*GFP*-*slt2*^T195V^ and pRS316-*GFP*-*slt2*^T195V Y198F^ were obtained with the same strategy and primers as pRS316-*slt2*^T195V^ and pRS316-*slt2*^T195V Y198F^, respectively, using pRS316-*GFP*-*SLT2* as the template.

### 4.2. Yeast Strains and Culture Conditions

All yeast strains used in this study are described in Table 2. The strains YMJ30 and YMJ31 were obtained by integrating the fragments *MKK1-6MYC* and *MKK2-6MYC* (from digestion of the plasmids pRS305-*MKK1-6MYC* and pRS305*-MKK2-6MYC* with *Cell*II and *Eco81*I, respectively) into the *MKK1* and *MKK2* loci of BY4741 *slt2Δ* (Y00993 strain). The strains YGGR32, YGGR33, and YGGR34 were constructed by amplification of the *slt2::NatMx4* cassette from the YSTH4 strain with the primers *SLT2*-pre121.5 and *SLT2*-post165.3, and subsequent integration into the BY4741 isogenic *kanMx4* deletion mutants *mkk1Δ, mkk2Δ*, and *msg5Δ* (Y02487, Y02212, and Y07373 strains, respectively).

Yeast cultures were grown in yeast extract–bacto-peptone–dextrose (YPD) medium or in selective synthetic dextrose media (SD) containing 2% glucose and the appropriate amino acids for plasmid selection. All strains were cultured at 30 °C unless stated otherwise. Experiments for analyzing Slt2 phosphorylation under stress were carried out by incubating cells overnight in synthetic dextrose (SD) media and refreshing them to an optical density (OD_600_) of 0.3 in YPD to achieve the mid-log phase. Cell cultures were then treated with 30 µg/mL of Congo Red (Merck, Darmstadt, Germany) and incubated for an additional 2 h. After that time, cells were collected and frozen immediately in liquid nitrogen for subsequent processing.

### 4.3. Preparation of Yeast Extracts and Immunoblotting Analysis

Yeast extracts were obtained by trichloroacetic acid precipitation as previously described [58]. Once solubilized, samples were boiled for 5 min and allowed to cool before loading. Samples to be analyzed by SDS–PAGE 10% 37.5:1 acrylamide:Bis–acrylamide gels (PanReac AppliChem ITW Reagents, Barcelona, Spain) were run at a constant voltage of 170 V until the bromophenol blue reached the bottom of the gel, and transferred to nitrocellulose membranes for immunoblotting (110 V for 70 min). Analysis of protein phosphorylation by Phos-tag was performed using Zn^2+^–Phos-tag SDS–PAGE in conjunction with a Bis–tris-buffered neutral-pH gel system, as described in [59]. We optimized several formulations and adapted several protocol steps to properly resolve different phosphorylation states, as indicated below. The resolving gel solution was composed of 8% 37.5:1 acrylamide:bis–acrylamide mixture, 350 mM Bis–Tris pH 6.8, 20 µM Phos-tag™ Acrylamide (Fujifilm Wako Chemicals Europe GmbH, Neuss, Germany), 40 µM ZnCl_2_, 0.1% tetramethylethylenediamine (TEMED), and 0.05% ammonium peroxysulfate (PSA). The resolving gel was poured into the cassette, overlayed with isopropanol, and polymerized at room temperature for 1 h. Before adding the stacking gel solution to the cassette, the resolving layer was rinsed five times with 350 mM Bis–Tris (pH 6.8). The stacking gel solution (4.5% 37.5:1 acrylamide:Bis–acrylamide, 350 mM Bis–Tris pH 6.8; 0.1% TEMED, and 0.05% PSA) was polymerized at room temperature for 1 h. Samples to be analyzed by Zn^2+^–Phos-tag SDS–PAGE were run at a constant current of 20 mA per gel for 150 min. Prior to transfer, the gel was immersed in transfer buffer containing 5 mM ethylenediaminetetraacetic acid (EDTA) (twice, for 10 min each). Next, the gel was immersed in blotting buffer without EDTA for 10 min. Proteins were then transferred to the membrane using the tank method in buffer containing 0.1% SDS overnight (18 h), at 20 V and 4 °C. After protein transfer, the membrane was washed in deionized water (four times, for 5 min each).

Both SDS–PAGE and Zn^2+^–Phos-tag SDS–PAGE gels were cast, run, and transferred using the 1.0-mm Bio-Rad Mini-PROTEAN gel system and a 0.45 µm nitrocellulose blotting membrane (GE Healthcare Life Sciences, Chicago, IL, USA). The resulting membranes were blocked by incubation in 5% non-fat dry milk diluted in phosphate-buffered saline (PBS) Tween 0.1% for 1 h at room temperature.

Membranes were probed with an appropriate primary antibody, all of which are listed in Table 3, and diluted in 1% non-fat dry milk in PBS Tween 0.1%.

After washing samples with PBS Tween 0.1% (five times, for 5 min each), membrane-bound immune complexes were detected by incubation with a 1:5000 dilution of an appropriate infrared dye-labeled secondary antibody—800 CW Goat anti-Rabbit IgG (926-32211), 680LT Goat anti-Rabbit IgG (926-68021), 800 CW Goat anti-Mouse IgG (926-32210), or 680LT Goat anti-Mouse IgG (926-68020) (LI-COR Biosciences, Lincoln, NE, USA). After washing as described above, immunoblots were analyzed with an infrared imaging system (Odyssey, LI-COR Biosciences).

### 4.4. Yeast Drop Dilution Growth Assays

To perform the growth assays, cultures were grown to saturation overnight in SD media, and adjusted to an OD_600_ of 0.5 in water. Tenfold serial dilutions of the cell suspensions were spotted onto agar plates containing YPD medium. When indicated, YPD plates were supplemented with a corresponding concentration of any of the following compounds: Congo Red (Merck); caffeine (Sigma-Aldrich); SDS (PanReac Applichem ITW Reagents); tunicamycin (Sigma-Aldrich); Calcofluor White (Sigma-Aldrich); or zymolyase 100T (MP Biomedicals™, Santa Ana, CA, USA). The plates were imaged after incubation at 30 °C (or 38 °C, when indicated) for 72 h.

### 4.5. Co-Purification Experiment with Dynabeads^TM^ Protein G

For protein extraction, cells were resuspended in cold lysis buffer (50 mM Tris/HCl (pH 7.5), 5 mM EDTA (pH 8), 150 mM NaCl, 50 mM NaF, 5 mM sodium pyrophosphate, 10% glycerol, 0.1% NP-40, 50 mM β-glycerol phosphate, and 1 mM sodium orthovanadate) supplemented with 1 mM phenylmethylsulfonyl fluoride (PMSF) and protease inhibitor mixture (Thermo Fisher Scientific, Waltham, MA, USA) and lysed using 0.75–1 mm diameter glass beads. The extracts were then adjusted to the same protein concentration and incubated with Dynabeads^TM^ Protein G (Thermo Fisher Scientific) coupled to the Mpk1-E9 antibody overnight at 4 °C. Beads were extensively washed with washing buffer (0.05% Tween 20 and 0.5 M NaCl in PBS) and protein complexes were eluted with elution buffer (50 mM glycine pH 2.8). Then, 5x SDS loading buffer was added; samples were boiled for 5 min, analyzed by SDS–PAGE, and immunoblotted as indicated above (4.3).

### 4.6. β-galactosidase Activity Assay

β-galactosidase activity was determined as described previously [36]. Briefly, extracts were obtained after breaking cells with 0.75–1 mm diameter glass beads; protein concentrations were determined by the Bradford method; and β-galactosidase activity was spectrophotometrically determined at 415 nm by using o-nitrophenyl-β-D-galactopyranoside (ONPG) as the substrate, expressed as nanomoles of o-nitrophenol/min/mg of total protein.

### 4.7. Microscopy Techniques

For GFP in vivo fluorescence microscopy, cells were collected by centrifugation at 2500 rpm and prepared for visualization. The microscope used was an Eclipse TE2000U (Nikon, Tokyo, Japan) with an appropriate set of filters. Digital images were acquired with an Orca C4742-95-12ER charge-coupled device camera (Hamamatsu, Hamamatsu city, Japan) and processed with the HCImage software (Hamamatsu).

## Figures and Tables

**Table 1:** Plasmids used in this study.

Plasmid	Source
pRS316	[54]
pRS316-*SLT2*	[55]
YCp50[*mpk1Y192F*]	[25]
YCp50[*mpk1T190A*]	[25]
pRS315	[54]
pRS315-*SLT2*	This study
pRS315-*slt2*^T190A^	This study
pRS315-*slt2*^Y192F^	This study
pRS316-*slt2*^T190A^	This study
pRS316-*slt2*^Y192F^	This study
pRS316-*slt2*^T190A Y192A^	This study
pRS316-*slt2*^ΔC374^	This study
pRS316-*slt2*^CD3^	This study
pHR3	[32]
pRS316-*slt2*^KD^	This study
pRS316-*slt2*^T195V^	This study
pRS316-*slt2*^Y198F^	This study
pRS316-*slt2*^T195V Y198F^	This study
YEp352GST	[30]
YEp352MSG5GST	[30]
Yp352MSG5^C319A^GST	[30]
p*MLP1-LacZ*	Dr. Javier Arroyo
pLA10	[56]
pRS316-6×His-Slt2	This study
pRS316-*GFP*-*SLT2*	This study
pRS316-*GFP*-*slt2*^T195V^	This study
pRS316-*GFP*-*slt2*^T195V Y198F^	This study
pRS305-*MKK1-6MYC*	[34]
pRS305*-MKK2-6MYC*	[34]

**Table 2:** Yeast strains used in this study.

Strain	Genotype	Reference
Y00993	BY4741; *slt2*Δ::*KanMx4*	Euroscarf
Y02487	BY4741; *mkk1*Δ:: *KanMx4*	Euroscarf
Y02112	BY4741; *mkk2*Δ:: *KanMx4*	Euroscarf
Y07373	BY4741; *msg5*Δ:: *KanMx4*	Euroscarf
BY4741 *slt2Δ*-RLM1Myc	BY4741; *slt2*Δ:: *KanMx4*; *RLM1- 6MYC:: HIS3*	[35]
YMJ30	BY4741, *slt*2Δ:: *KanMx4*, *MKK1*::*6MYC*::*LEU2*	Dr. Jiménez-Sánchez
YMJ31	BY4741, *slt*2Δ:: *KanMx4*, *MKK2*::*6MYC*::*LEU2*	Dr. Jiménez-Sánchez
YSTH4	Y3656; *slt2:: NatMx4*	[57]
YGGR32	BY4741, *mkk1*Δ:: *KanMx4*, *slt2*:: *NatMx4*	This study
YGGR33	BY4741, *mkk2*Δ:: *KanMx4*, *slt2*:: *NatMx4*	This study
YGGR34	BY4741, *msg5*Δ:: *KanMx4*, *slt2*:: *NatMx4*	This study

**Table 3:** Primary antibodies used in this study.

Name	Catalog Number	Species	Dilution Factor ^a^	Manufacturer
Mpk1 (E9)	sc-133189	Mouse mC	WB: 1:1000, IP: 1:20	Santa Cruz, Inc
Phospho-p44/42 MAPK (Erk1/2) (Thr202/Tyr204)	4370	Rabbit mC	WB: 1:1000	Cell signaling
Phospho-p44/42 MAPK (Erk1/2) (Thr202/Tyr204)	4377	Rabbit mC	WB: 1:250	Cell signaling
Diphosphorylated ERK-1&2	M8159	Mouse mC	WB: 1:1000	SIGMA
GST	sc459	Rabbit pC	WB: 1:1000	Santa Cruz, Inc.
Myc 4A6	05-724	Mouse mC	WB: 1:1000	Merck Millipore
C-Myc	PLA0001	Rabbit pC	WB: 1:1000	Sigma-Aldrich
Actin C4	69100	Mouse mC	WB: 1:1000	MP Biomedicals

^a^ Applications: WB—Western blotting and IP—immunoprecipitation.

## Data Availability

The data that support the findings of this study are available from the corresponding author upon reasonable request.

## Supplementary Materials

Supplementary Materials can be found at https://www.mdpi.com/1422-0067/22/3/1110/s1.

## Abbreviations

Caf	Caffeine
CD	Common docking
CFW	Calcofluor White
CR	Congo Red
CWI	Cell wall integrity
DSP	Dual-specificity phosphatases
EDTA	Ethylenediaminetetraacetic acid
ERK	Extracellular signal-regulated kinase
GFP	Green fluorescent protein
GST	Glutathione S-transferase
IB	Immunoblotting
IP	Immunoprecipitation
JNK	c-Jun N-terminal kinase
KD	Kinase dead
MAPK	Mitogen-activated protein kinase
MAPKKK/MAP3K	MAPK kinase kinase
MAPKK/MAP2K/MEK	MAPK kinase
MKP	MAPK phosphatase
mC	Monoclonal
OD	Optical density
ONPG	o-nitrophenyl-β-D-galactopyranoside
PAGE	Polyacrylamide gel electrophoresis
PBS	Phosphate-buffered saline
pC	Policlonal
PMSF	Phenylmethylsulfonyl fluoride
Pro	Proline
PSA	Ammonium peroxysulfate
PTMs	Post-translational modifications
SD	Synthetic dextrose
SDS	Sodium dodecyl sulfate
Ser	Serine
TEMED	tetramethylethylenediamine
Thr	Threonine
Tuni	Tunicamycin
Tyr	Tyrosine
WB	Western blotting
WT	Wild type
Zym	Zymolyase
