# Supplementary material for: Differential Role of Threonine and Tyrosine Phosphorylation in the Activation and Activity of the Yeast MAPK Slt2
Source: Int J Mol Sci. 2021 Jan 23;22(3):1110. doi: 10.3390/ijms22031110 (PMC7866135; doi:10.3390/ijms22031110)
Supplement: Supplementary file 1 [file ijms-22-01110-s001.pdf]

Table S1. Oligonucleotides used in this study.

Name	Primer
SLT2-5	5′-CAATTCTGGGAGATGGCTG-3′
SLT2-3	5′-CAAACTTCCGCGGAGTACG-3′
SLT2-MNP5	5′-CGAAAACAGTCAATTTTTGGCAGAGGCCGTGGCCACTAGATGG-3′
SLT2-MNP3	5′-CCATCTAGTGGCCACGGCCTCTGCCAAAAATTGACTGTTTTCG-3′
SLT2-MSTOP5	5′-GAAGAGCAAAGGCAATTATAATAACAGCAGCAGCAACAGC-3′
SLT2-MSTOP3	5′-GCTGTTGCTGCTGCTGTTATTATAATTGCCTTTGCTCTTC-3′
SLT2-CD5	5′-TACTTGTCTATATGGCATAATCCAGCTAACAATCCTGTGTGTAGTGAAAAAT-3′
SLT2-CD3	5′-ATTTTTCACTACACACAGGATTGTTAGCTGGATTATGCCATATAGACAAGTA-3′
SLT2-T195V5	5′-CGGAGTACGTGGCCGTTAGATGGTATAGAGCTCCGG-3′
SLT2-T195V3	5′-CCGGAGCTCTATACCATCTAACGGCCACGTACTCCG-3′
SLT2-Y198F5	5′-CGGAGTACGTGGCCACTAGATGGTTTAGAGCTCCGG-3′
SLT2-Y198F3	5′-CCGGAGCTCTAAACCATCTAGTGGCCACGTACTCCG-3′
SLT2-T195VY198F5	5′-CGGAGTACGTGGCCGTTAGATGGTTTAGAGCTCCGG-3′
SLT2-T195VY198F3	5′-CCGGAGCTCTAAACCATCTAACGGCCACGTACTCCG-3′
SLT2-pre121.5	5′-CGAACTGTGCATTCAGTCAG-3′
SLT2-post165.3	5′-CGAGCTACAACAAGAGCAC-3′
SLT2-preSalI.5	5′-GTGAGTTACCTCACTCATTAG-3′
SLT2-postEcoRI.3	5′-ACGACGTTGTAAAACG-3′
SLT2-*Cla*I6×His*Mlu*I.5	5′-ATCGATATGTCTCATCATCACCACCATCATACGCGTATGGCTGATAAGATAGAGAG-3′
SLT2-*Cla*I6×IHis*Mlu*I.3	5′-ACGCGTATGATGGTGGTGATGATGAGACATATCGATCTCCCAGAATTGTTATACAC-3′
GFP-5	5′- CCATCGATATGAGTAAAGGAGAAGAAC-3′
GFP-3	5′-CGACGCGTTTTGTATAGTTCATCCATGC-3′

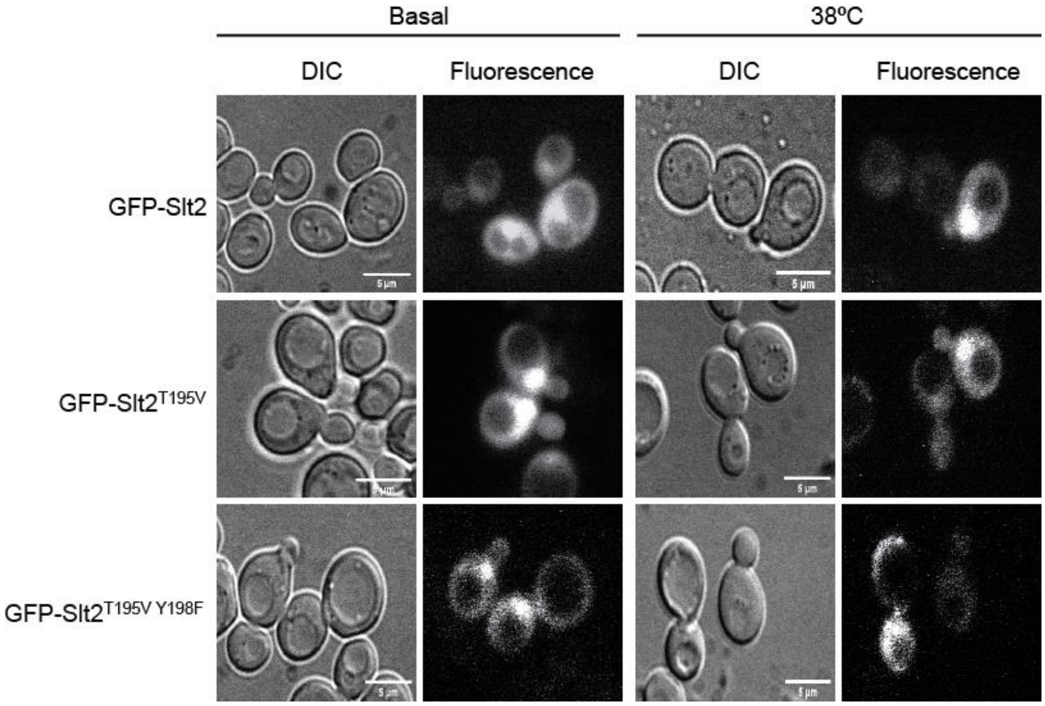

Figure S1. Slt2 localization is not affected by T195V and Y198F mutations. DIC (Differential Interference Contrast) and fluorescence microscopy images of BY4741 *slt2Δ* (Y0093 strain) cells expressing GFP-Slt2, GFP-Slt2^{T195V} or GFP-Slt2$^{T195V\ Y198F}$, in basal conditions or under heat treatment (38ºC) for 2h. Scale bar = 5μM.
